# Transcriptome analysis clarified genes involved in resistance to *Phytophthora capsici* in melon

**DOI:** 10.1371/journal.pone.0227284

**Published:** 2020-02-12

**Authors:** Pingyong Wang, Haibo Wu, Guangwei Zhao, Yuhua He, Weihu Kong, Jian Zhang, Shuimiao Liu, Mengli Liu, Keyun Hu, Lifeng Liu, Yongyang Xu, Zhihong Xu

**Affiliations:** 1 Zhengzhou Fruit Research Institute, Chinese Academy of Agricultural Sciences, Zhengzhou, Henan Province, China; 2 Hainan Sanya Trial Center for Crops Breeding of Xinjiang Academy of Agricultural Sciences, Sanya, Hainan Province, China; National Institute of Technology Rourkela, INDIA

## Abstract

*Phytophthora blight* caused by *Phytophthora capsici* is a devastating disease for melon plant. However, the underlying resistance mechanisms are still poorly understood. In this study, the transcriptome differences between the resistant ZQK9 and susceptible E31 at 0, 3, and 5 days post-inoculation (dpi) were identified by RNA-seq. A total of 1,195 and 6,595 differentially expressed genes (DEGs) were identified in ZQK9 and E31, respectively. *P*. *capsici* infection triggered massive transcript changes in the inoculated tissues. Genes related to plant defense responses were activated, which was reflected by a lot of up-regulated DEGs involved in pathogenesis-related (PR) genes, hormones biosynthesis and signal transduction, secondary metabolites biosynthesis and cell wall modification in resistant ZQK9. The dataset generated in this study may provide a basis for identifying candidate resistant genes in melon against *P*. *capsici* and lay a foundation for further research on the molecular mechanisms.

## Introduction

Melon (*Cucumis melo* L.) is an economically important fruit worldwide and plays an important role in promoting income of growers, as well as improving the diet structure of local residents. However, the expansion of melon growing areas and its intensive year-round cultivation result in the occurrence of serious biotic challenges, especially soil-borne diseases, which lead to substantial decreases in melon yield and quality [1].

*Phytophthora blight*, a soil-borne disease caused by *Phytophthora capsici*, is a devastating disease for melon. It has spread over the cucurbit growing areas, especially in tropical and subtropical regions. *P*. *capsici* can infect host plants at any growth stage, causing necrosis in the roots, stems, leaves, crowns, and fruits [2–4]. Infected plants rarely overcome the disease and usually die off quickly. High temperature and humidity conditions accelerate the spread of this disease. Thus, there is a great need for prevention in protected cultivation areas.

However, *P*. *capsici* is difficult to control once it has infected the soil, as the pathogen can overwinter with the type of oospores or mycelium in infected soil and plant residue. Oospores can endure extreme environmental conditions, such as desiccation and cold temperature, and can survive in the soil for many years, even without a host plant [5,6]. Traditionally, agricultural and chemical prevention measures are utilized to reduce the damage caused by *P*. *capsici*. Along with the enhancement of food safety and environmental protection awareness and requests for high-quality fruit products, it has become more and more important to develop disease-resistant varieties. However, most of the present melon cultivars are susceptible to *P*. *capsici*, and very few resistant melon germplasms were reported to possess high resistance level, which immensely restrict the resistance inheritance research [7,8]. To our knowledge, no disease-resistant gene has been mapped or cloned yet in melon.

Analysis of global gene expression is one means to explore the molecular basis of interactions between host plants and *Phytophthora* pathogens, particularly with respect to mechanisms of resistance and the basal defense response. Naveed et al. analyzed the transcriptome of two wild tomato accessions in response to *P*. *parasitica* infection and found that some genes annotated as protease inhibitors, chitinases, defensin and PR-1 were highly induced in resistant accession [9]. Mansfeld et al. focused on cucumber fruit peel genes responsible for age-related resistance to *P*. *capsici* and reported that numerous genes involved in the synthesis and decoration of flavonoid and terpenoid were up-regulated in resistant age peels at 16 days post pollination [10]. In tobacco leaves infected by *P*. *parasitic*, Shen et al. identified 8989 differentially expressed genes (DEGs), which included a larger number of up-regulated JA and ET signaling genes, receptor-like kinase, PR genes, and transcription factors [11]. Therefore, comparing responses to *P*. *capsici* infection in resistant and susceptible plant lines is critical for understanding the defense mechanisms. RNA-seq technology provides a far more precise measurement of transcript levels to identify the primary changes in gene expression.

In our previous study, a melon line ZQK9 was identified to be highly resistant to *P*. *capsici*, while an inbred line E31 was found to be highly susceptible. In this study, RNA-seq technology was conducted to analyze the transcriptome changes in the roots of ZQK9 and E31 in response to *P*. *capsici* at 0, 3, and 5 days post-inoculation (dpi). Identification of DEGs associated with the defense response will increase our understanding of the molecular basis for resistance to *P*. *capsici* in melon, as well as contribute to the melon cultivar improvement.

## Materials and methods

### Plant materials

The resistant line ZQK9 (Genbank number: ZTG00632) was obtained from the National Mid-term Genebank for Watermelon and Melon, the Zhengzhou Fruit Research Institute, Chinese Academy of Agricultural Sciences. It exhibited high resistance to *P*. *capsici* with no symptoms after inoculation. The inbred line, E31, was bred by Zhengzhou Fruit Research Institute of Chinese Academy of Agricultural Sciences and used as the highly susceptible line. The plastic bowls measuring 9×7×8 cm were filled with mixed peat and vermiculite with the volume of 1:1. Seeds of the resistant and susceptible lines were sown in plastic bowls with one seed per bowl. Plants were cultivated in a growth cabinet under a photoperiod of 16 h light/8 h dark and air temperatures of 28/20°C. Seedlings with two fully expanded true leaves were used for *P*. *capsici* inoculation.

### *P*. *capsici* inoculation and sample collection

*P*. *capsici* was isolated from infected melon plants collected from Hainan province, China, and verified through morphological identification and PCR-based detection following previously described protocols [12]. Purified hypha of *P*. *capsici* was transferred on PDA medium in petri dishes (9 cm diameter) and cultivated at 25°C in the dark for 5 d. Then, a piece of hypha from the edge of the *P*. *capsici* colony was transferred to carrot agar medium for spore production [13]. The pathogen was cultivated at 25°C in the dark for 5 d and then 12 h light/12 h dark photoperiod for 5–7 d. Mycelia in each petri dish was soaked with sterilized water and cultivated at 4°C for 40–60 min. Then, petri dishes were placed under light at 25°C for 40 min for zoospore release from broken sporangia. Zoospore suspension was sucked out by a pipette and filtered with two layers of gauze. The concentration of zoospore suspension was counted with a hemacytometer and diluted to 1×10^6^ mL^-1^ prior to inoculation.

Before inoculation, seedlings were watered until the soil moisture content was saturated. One milliliter of zoospore suspension was released onto the soil surface around each seedling’s primary root. ZQK9 and E31 plant roots were sampled at 0 dpi (control treatment) and again at 3 and 5 dpi for sequencing. Samples were referred to as R0, R3, and R5 for the resistant line, and S0, S3, and S5 for the susceptible line. The whole seedling with soil was taken out from the plastic bowl, and soaked in distilled water. By gently shaking, the soil was removed and the whole root was washed clean. Then the whole root was wrapped in tinfoil and immediately snap-frozen in liquid nitrogen and stored at -80°C. At each time point, the roots of 9 plants (three biological replications, three plants for each replicate) were sampled and pooled for RNA extraction.

### RNA isolation and library construction for sequencing

Total RNA was extracted using TRIzol reagent (Invitrogen, CA, USA). The purity of total RNA was detected by a NanoPhotometer (Life Technologies, CA, USA) and 1% agarose gel electrophoresis. RNA concentration and integrity value (RIN) was checked using an Agilent 2100 Bioanalyzer (Agilent, CA, USA). Total amount of 2 μg RNA per sample was used for the RNA sample preparations. Sequencing libraries were generated using the E7530L NEBNext® Ultra^™^ RNA Library Prep Kit for Illumina® (NEB, MA, USA) and index codes were added to attribute sequences to each sample. Briefly, mRNA was purified from total RNA using poly-T oligo-attached magnetic beads. Fragmentation was carried out using divalent cations under elevated temperature in NEBNext First Strand Synthesis Reaction Buffer (5X). First strand cDNA was synthesized using random hexamer primer and RNase H. Second strand cDNA synthesis was subsequently performed using buffer, dNTPs, DNA polymerase I and RNase H. The library fragments were purified with QiaQuick PCR kits (QIAGEN, Hilden, Germany) and elution with EB buffer, then terminal repaired, A-tailing and adapter added were implemented. The aimed products (about 350 bp) were retrieved and PCR was performed, then the library was completed. Collectively, 18 libraries consisting of root samples from ZQK9 and E31 at 0, 3, and 5 dpi were constructed and sequenced by an Illumina HiSeq^TM^ 4000 platform (Illumina, CA, USA).

### Sequence assembly, annotation, and DEG analysis

Raw reads were filtered by removing the adaptor and low-quality reads for further analysis. The remaining clean reads were mapped to the reference melon (DHL92) genome (Version 3.5.1, http://cucurbitgenomics.org/) using TopHat and assembled by Cufflinks software [14–16]. Fragments per Kilobase per Million (FPKM) values of all genes in each sample were calculated to quantify gene expression. DEGs between two samples were identified by DEGseq software with the threshold, |log_2_Ratio| ≥ 1 and *q* < 0.05, and compared to public databases for functional annotations, including the National Center for Biotechnology Information (NCBI), Uniprot, Gene Ontology (GO), and Kyoto Encyclopedia of Genes and Genomes (KEGG), with the threshold, *q* < 0.05 and False Discovery Rate (FDR) ≤ 0.05 [17]. If a given gene matched multiple protein sequences, the protein with the highest degree of similarity was considered the optimal annotation. To infer transcriptional changes over time under *P*. *capsici* infection, DEGs were identified by comparing expression levels at 3 and 5 dpi to expression levels at 0 dpi in both ZQK9 and E31. For convenience, DEGs exhibiting higher expression levels at 3 and 5 dpi than at 0 dpi were designated as “up-regulated” (log_2_Ratio ≥ 1, *q* < 0.05), while those that exhibited lower expression levels were designated as “down-regulated” (log_2_Ratio ≤ -1, *q* < 0.05).

### qRT-PCR analysis

To validate the RNA-seq results, quantitative real-time PCR (qRT-PCR) was conducted on 14 DEGs that may be involved in the resistance to *P*. *capsici*. First strand cDNA was synthesized using a PrimeScript^™^ RT reagent Kit with gDNA Eraser (Takara, Dalian, China). PCR reactions were performed on a Roche LightCycler® 480 RT-PCR System (Roche Diagnostics, Rotkreuz, Switzerland) using the RR420A SYBR Premix Ex Taq^™^ Kit (Takara, Dalian, China). Primers were designed using Primer Premier v5.0 software (Premier Biosoft International, CA, USA) and listed in S1 Table. Gene expression analysis was conducted on all ZQK9 and E31 samples obtained at 0, 3, and 5 dpi. Three independent biological and technical replicates were set up. The melon actin gene, *MELO3C023264*, was used as an internal control gene. Expression levels were analyzed using the 2^-ΔΔCt^ method [18].

### Histological study of ZQK9 and E31

Seedlings of ZQK9 and E31 were sampled at 0, 3, and 5 dpi and fixed in formalin-aceto-alcohol fixative (FAA; 5 mL formalin + 5 mL acetic acid + 90 mL 70% ethanol) for histological study [19]. In each case, three seedlings were sampled and treated. Primary root of 1 cm length away from radicle/hypocotyl interface was selected for dissection. The root segments were immersed in FAA at 4°C for at least 24 h, then dehydrated in an ethanol series (30% ethanol 12 h, 50% ethanol 8 h, 75% ethanol 4 h, 85% ethanol 2 h, 90% ethanol 2 h, 95% ethanol 1 h, and 100% ethanol 1 h) and embedded in paraffin. Slices (5 μm thickness) were serially cut using a Leica microtome. The longitudinal sections of ZQK9 and E31 roots at 0, 3, and 5 dpi were used for periodic acid-schiff (PAS) stain [20].

## Results

### Comparative analysis of ZQK9 and E31 infected by *P*. *capsici*

**Fig 1:**
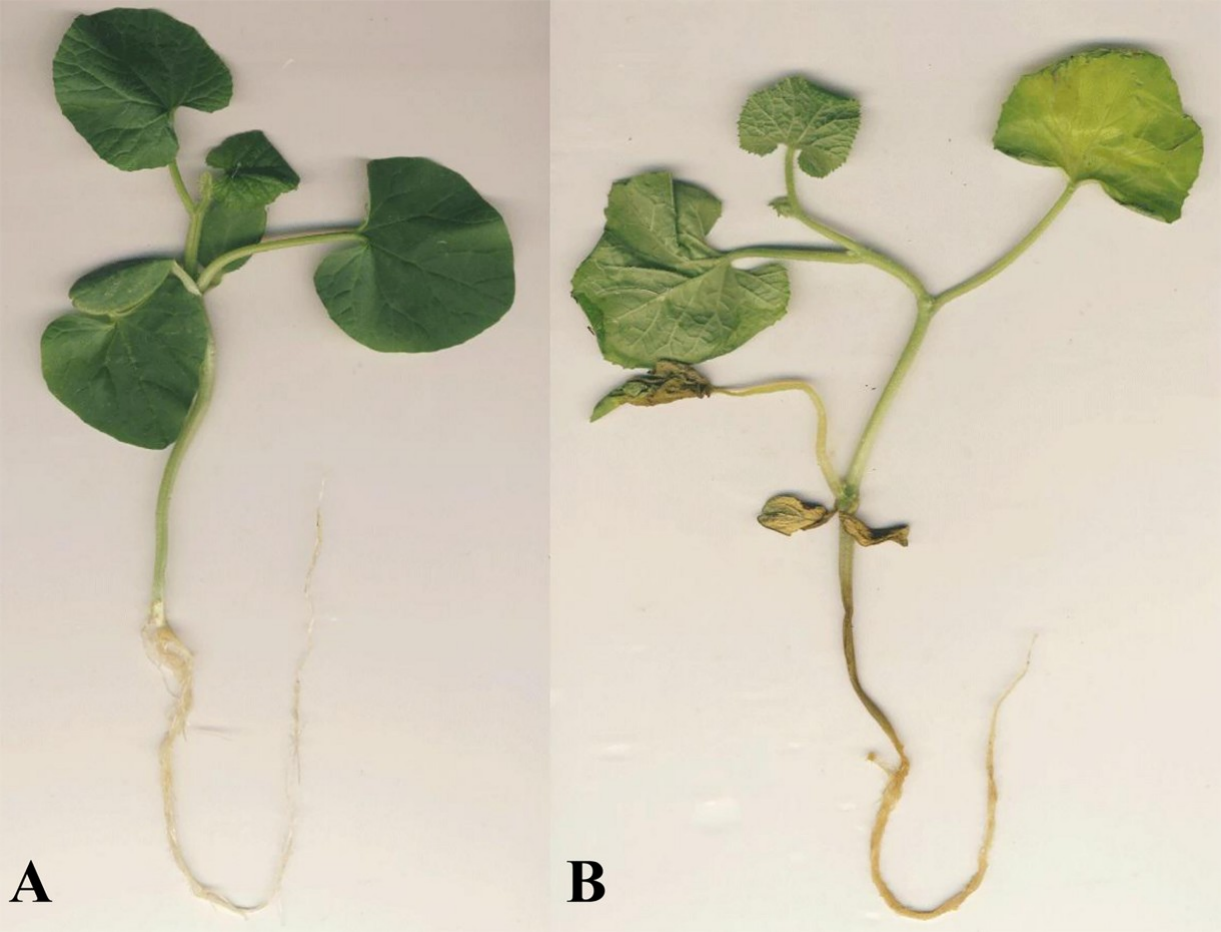
Disease symptoms of ZQK9(A) and E31 (B) at 5 dpi.

**Fig 2:**
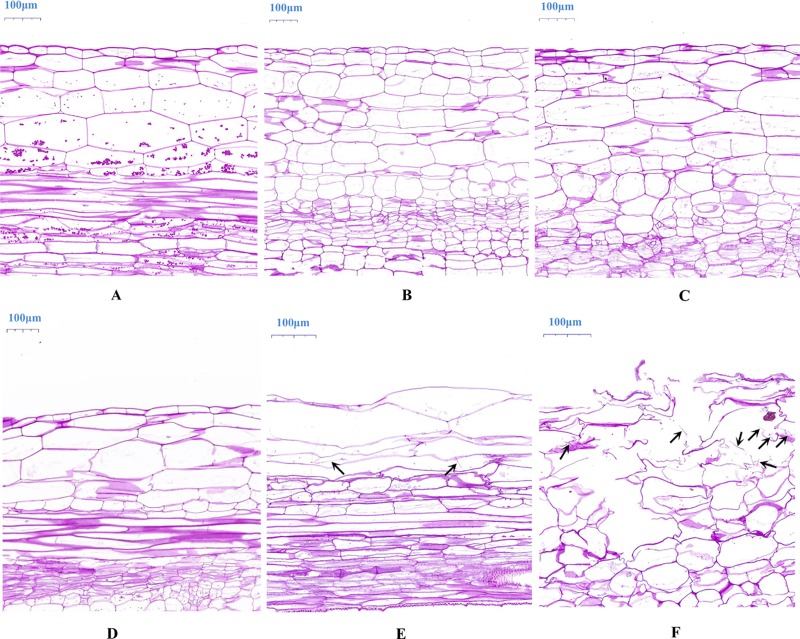
Histological images of ZQK9 and E31 roots inoculated by *P*. *capsici*. (A-C) longitudinal sections of ZQK9 root tissues through periodic acid-schiff (PAS) stain at 0, 3, and 5 dpi. (D-F) longitudinal sections of E31 root tissues through PAS stain at 0, 3, and 5 dpi. Arrows indicated suspected hyphae observed in E31 at 3 and 5 dpi, respectively.

To compare the different responses to *P*. *capsici*, the roots of ZQK9 and E31 were inoculated with zoospore suspension when the first two true leaves were fully expanded. At 3 dpi, water-soaked lesions with slight brown color appeared on E31 primary root. At 5 dpi, obvious necrosis spots could be observed on the root and hypocotyl of E31 (Fig 1B). In contrast, ZQK9 did not exhibit any obvious disease symptoms at both time points (Fig 1A). Primary root of 1 cm length away from radicle/hypocotyl interface was selected for dissection and PAS stain. The result showed root tissues of E31 were colonized by *P*. *capsici*. The cortical parenchyma cells became shrunken and partly deformed at 3 dpi (Fig 2E), and plasma membranes of infected cells were ruptured and progressively plasmolysed at 5 dpi (Fig 2F) compared to the root tissues at 0 dpi (Fig 2D). In the root of ZQK9, hyphae rarely progressed in tissues, and the plant cell structure remained intact (Fig 2A, 2B and 2C).

### RNA sequencing and reads alignment

Eighteen libraries were sequenced, generating more than 806 million raw reads with an average of 44.8 million in each sequencing library. The proportion of clean reads ranged from 95.93% to 97.18%. More than 79.00% of clean reads could be mapped to the reference genome (S2 Table). Among the mapped reads, more than 99.00% were uniquely mapped (S3 Table), and more than 85.18% of these mapped reads were aligned to exon regions (S4 Table). All of the raw data were deposited in the National Center for Biotechnology Information Sequence Read Archive (NCBI SRA) under the accession number PRJNA544826.

### DEGs response to *P*. *capsici*

**Fig 3:**
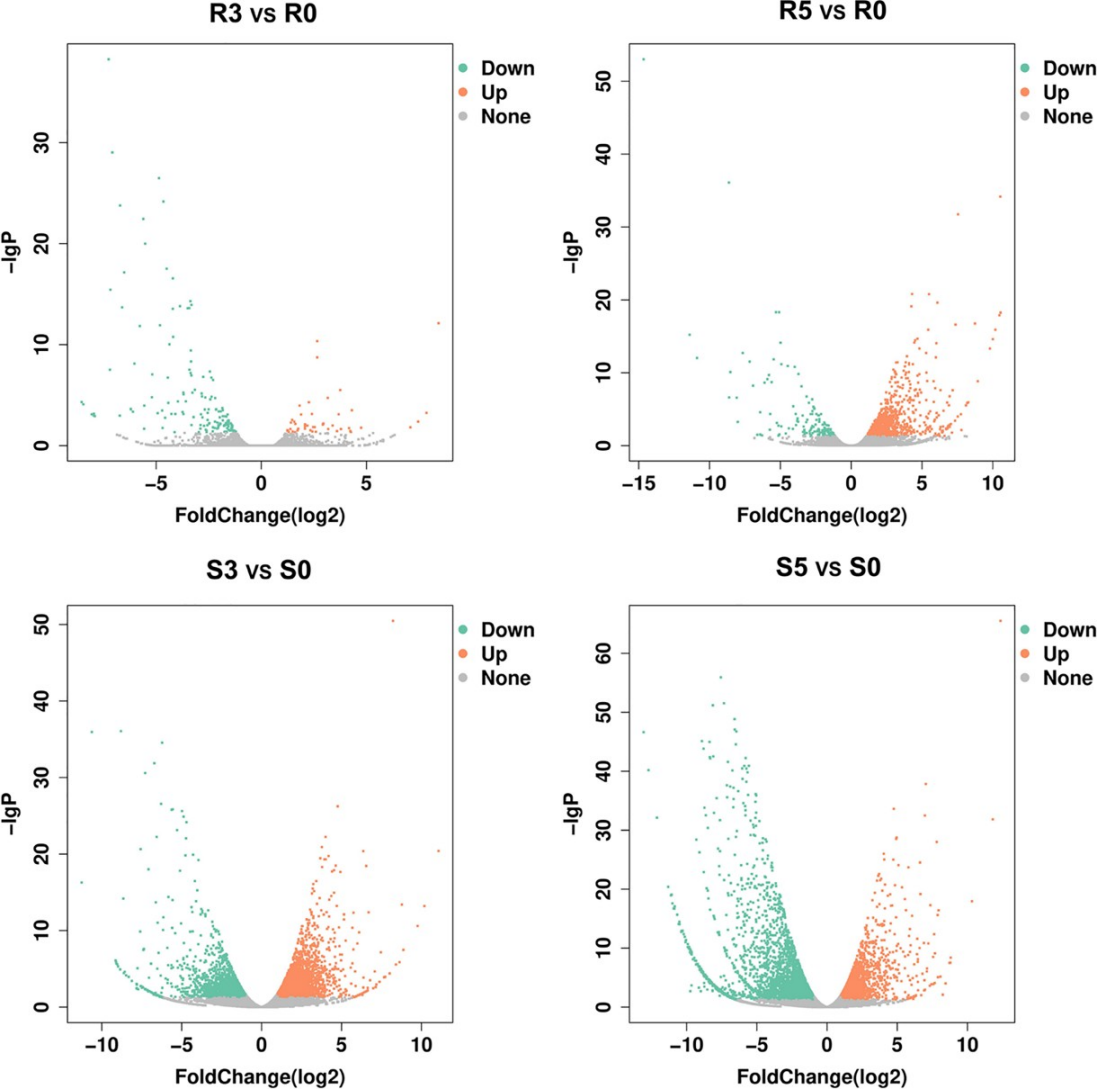
DEGs in the pairwise R3 vs R0, R5 vs R0, S3 vs S0, S5 vs S0. R3 vs R0 and R5 vs R0 indicated DEGs at 3 and 5 dpi in ZQK9, respectively. S3 vs S0 and S5 vs S0 indicated DEGs at 3 and 5 dpi in E31, respectively. The x-axes indicated fold change values (*q* < 0.05) and the y-axes represented the statistical significance of differences of gene expression. DEGs were shown in red and green dots, indicating up-regulated and down-regulated genes, respectively.

The global transcriptomes of ZQK9 and E31 at 3 and 5 dpi compared with control treatment were analyzed with the criteria of |log_2_Ratio| ≥ 1 and *q* < 0.05, and visualized by volcano plot (Fig 3). A total of 1,195 and 6,595 DEGs were identified in the inoculated roots of ZQK9 and E31, respectively. At 3 dpi, 42 DEGs were up-regulated and 136 DEGs were down-regulated in ZQK9. In E31, 2,143 DEGs were up-regulated, while 1,649 DEGs were down-regulated. At 5 dpi, 927 DEGs were up-regulated and 137 DEGs were down-regulated in ZQK9. In E31, 1,895 DEGs were up-regulated and 2,747 DEGs were down-regulated.

### Expression pattern of DEGs during the *P*. *capsici* infection

**Fig 4:**
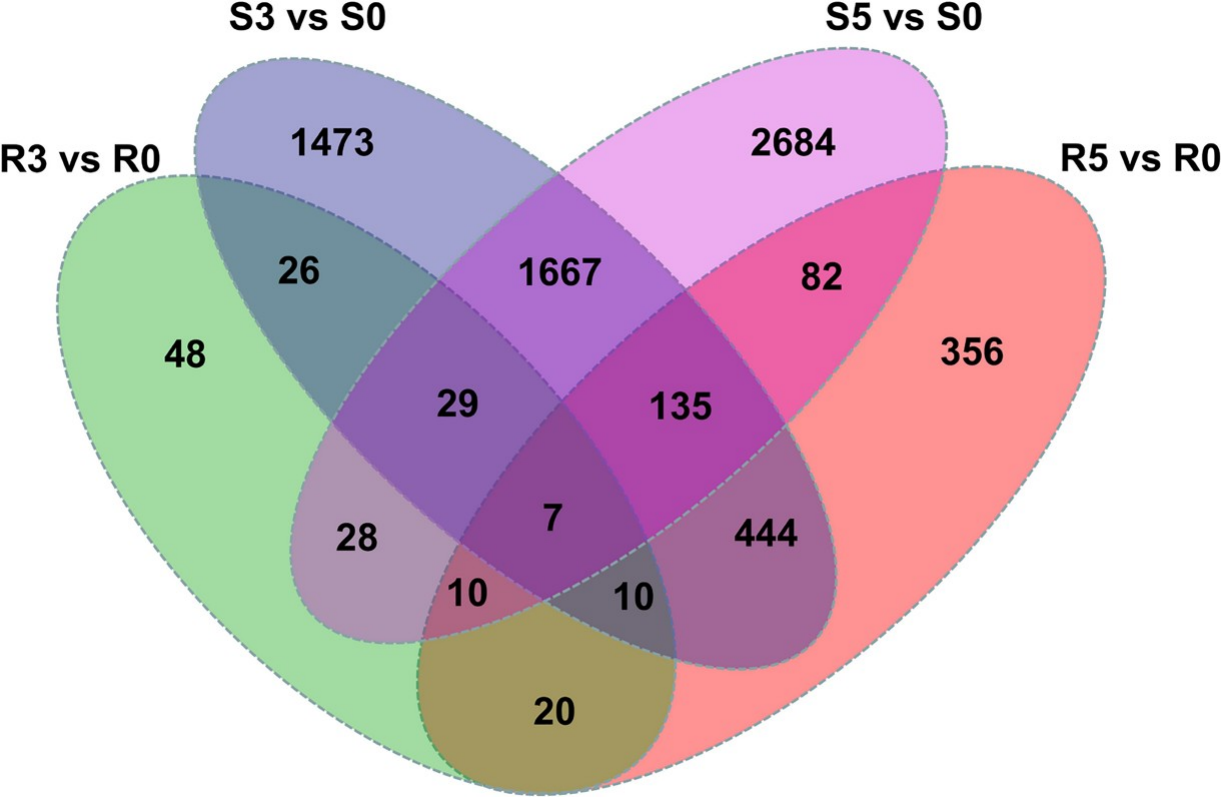
Venn diagram comparing the distribution of DEGs between ZQK9 and E31 at 3 and 5 dpi. R3 vs R0 and R5 vs R0 indicated the number of DEGs at 3 and 5 dpi in ZQK9, respectively. S3 vs S0 and S5 vs S0 indicated the number of DEGs at 3 and 5 dpi in E31, respectively.

Venn diagram was used to analyze the distribution of DEGs between ZQK9 and E31 at two infection stages (Fig 4). In ZQK9, 47 genes were differentially expressed at both infection stages compared to the control treatment, while 1,838 DEGs in E31 were differentially expressed at both stages. At 3 dpi, 72 DEGs were co-modulated in both ZQK9 and E31. At 5 dpi, 234 DEGs were co-modulated in both lines. 7 DEGs were differentially expressed in ZQK9 and E31 at both 3 and 5 dpi.

**Fig 5:**
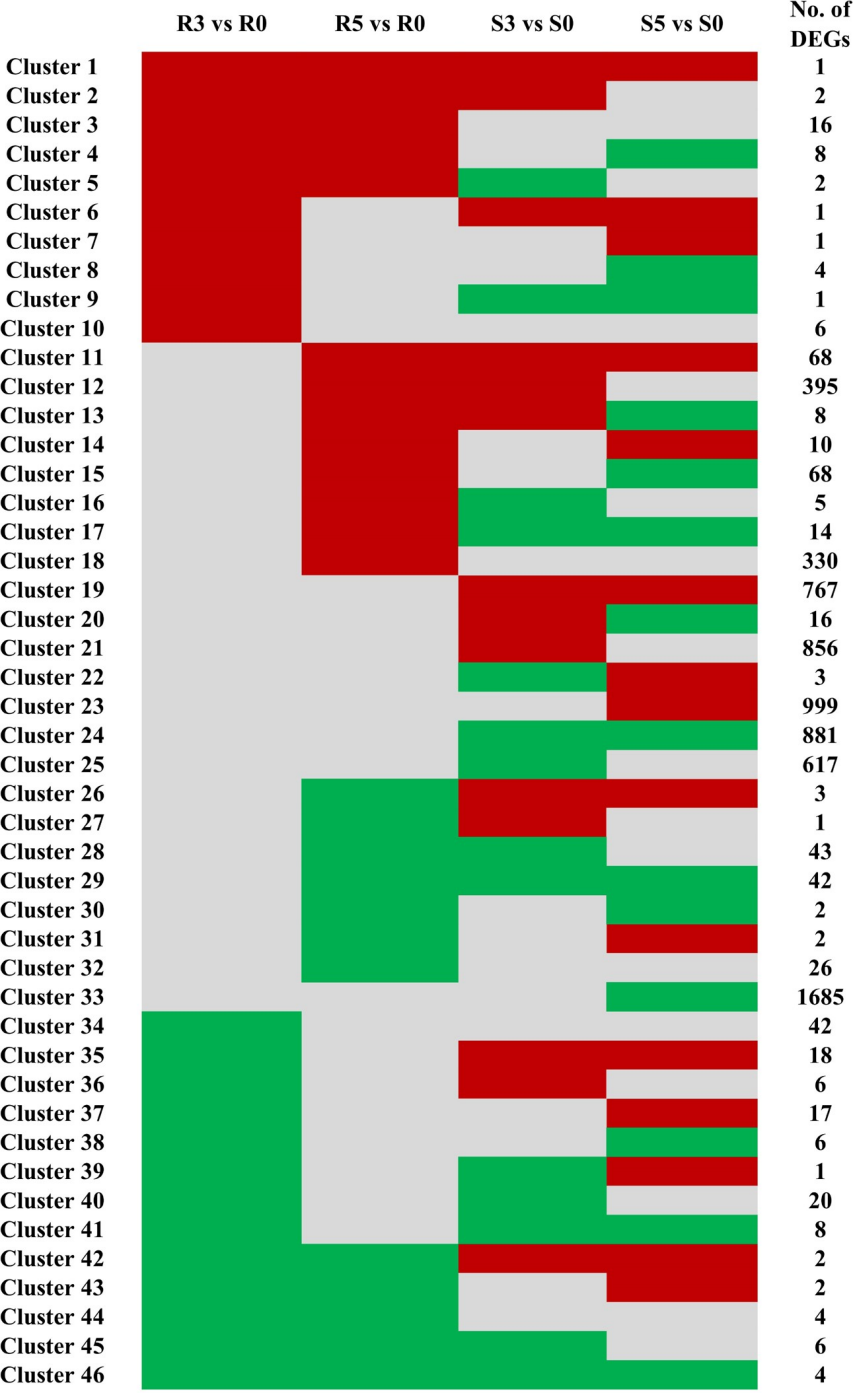
Clustering analysis of the DEGs with similar expression patterns in ZQK9 and E31 at 3 and 5 dpi. Up-regulation and down-regulation were represented by red and green shading, respectively. Gray shading indicated non-modulation. R3 vs R0 and R5 vs R0 were the DEGs at 3 and 5 dpi in ZQK9, respectively. S3 vs S0 and S5 vs S0 were the DEGs at 3 and 5 dpi in E31, respectively.

Clustering analysis of the DEGs at 3 and 5 dpi in ZQK9 and E31 was performed by extracting the genes with similar expression patterns (Fig 5). There were 46 clusters representing different gene expression patterns. Cluster 33 was the largest with 1,685 down-regulated DEGs, followed by cluster 23 with 999 up-regulated DEGs. All DEGs in these two clusters were only induced in E31 at 5 dpi. Cluster 1 included only one DEG *MELO3C026183*, which was annotated as a bidirectional sugar transporter SWEET12 and up-regulated in both ZQK9 and E31 at 3 and 5 dpi. Comparatively, all four DEGs in cluster 46, *MELO3C008050*, *MELO3C014099*, *MELO3C015081*, and *MELO3C026570*, were down-regulated in both ZQK9 and E31 at both infection stages. The DEGs in clusters 3, 4, and 5 were up-regulated in ZQK9 at both infection stages, with a highly expressed DEG, *MELO3C004387*, which was annotated as a pathogenesis-related protein PR-4B. *MELO3C016405* in cluster 4, which was annotated as a peroxidase 72-like gene, was up-regulated in ZQK9 but down-regulated in E31. The DEGs in clusters 8, 9, and 10 were up-regulated in ZQK9 at 3 dpi, including one thaumatin-like protein gene *MELO3C009903* in cluster 8 and one ethylene-responsive transcription factor (EFR) gene *MELO3C013593* in cluster 10. In cluster 14, there were 10 DEGs strongly up-regulated at 5 dpi in both lines, including one cellulose synthase-like gene *MELO3C006455*. In clusters 15, 16, 17, and 18, there were a total of 417 DEGs up-regulated in ZQK9 at 5 dpi. In cluster 42, the peroxidase 5-like gene *MELO3C021297* was down-regulated in ZQK9 but up-regulated in E31 at both infection stages (S5 Table). These specific DEGs were promising candidates for melon resistance to *P*. *capsici*.

### Functional annotation of DEGs

#### GO classification of DEGs

GO enrichment analysis was conducted on the four groups of DEGs (i.e., R3 vs R0, R5 vs R0, S3 vs S0, and S5 vs S0). Among the four groups, there were 148 (83.15%), 843 (79.23%), 2,952 (77.87%), and 3,721 (80.16%) DEGs that were assigned to at least one GO term. These GO terms included three classes: biological processes, cellular components, and molecular function. More assigned GO terms were covered in biological processes and cellular components than in molecular function categories. Although the dominant subcategories were similar in both lines at both infection stages, individual DEGs consisted in the same enriched subcategories were diverse.

At 3 dpi, no defense-related GO term was significantly enriched with the threshold of *q* < 0.05 and FDR ≤ 0.05 in ZQK9. While, in E31, response to stress (GO:0006950), response to stimulus (GO:0050896), defense response (GO:0006952), cellular response to reactive oxygen species (GO:0034614) and 29 other disease-resistance GO terms were significantly enriched.

In ZQK9 at 5 dpi, all 21 significantly enriched GO terms were related to defense response, including secondary metabolite biosynthetic process (GO:0044550), cellular response to oxidative stress (GO:0034599), response to reactive oxygen species (GO:0034614), response to extracellular stimulus (GO:0009991), lignin catabolic process (GO:0046274), etc. In E31, the significantly enriched GO terms that were related to defense response included cell wall organization or biogenesis (GO:0071554), cell wall organization (GO:0071555), external encapsulating structure organization (GO:0045229), and plant-type cell wall organization or biogenesis (GO:0071669), etc., which indicated the cell architecture of E31 were damaged by *P*. *capsici* infection (S6 Table).

#### Metabolic pathway of DEGs by KEGG analysis

KEGG analysis was performed to characterize the pathway enrichment of the identified DEGs. In ZQK9, there were 218 DEGs enriched in 32 pathways. Comparatively, there were 816 DEGs in E31 enriched in 85 pathways. Among these, 273 DEGs (44 in ZQK9, 253 in E31 and 24 in both lines) were enriched in 10 representative defense-related pathways, including plant-pathogen interactions, plant hormone signal transduction, phenylpropanoid biosynthesis, oxidative phosphorylation, brassinosteroid biosynthesis, terpenoid backbone biosynthesis, flavonoid biosynthesis, isoquinoline alkaloid biosynthesis, glutathione metabolism, cutin, suberine, and wax biosynthesis (Table 1, S7 Table).

**Table 1:** Significantly enriched KEGG pathways of differentially expressed genes induced by *P*. *capsici* infection.

Pathway	Map ID	R3 vs R0		R5 vs R0		S3 vs S0		S5 vs S0	
		Up	Down	Up	Down	Up	Down	Up	Down
Plant-pathogen interaction	map04626	0	1	4	0	16	13	14	19
Plant hormone signal transduction	map04075	0	1	5	3	29	26	24	35
Phenylpropanoid biosynthesis	map00940	1	3	12	0	12	12	9	28
Cutin, suberine and wax biosynthesis	map00073	0	0	3	0	0	0	0	6
Oxidative phosphorylation	map00190	1	0	1	0	3	16	3	17
Brassinosteroid biosynthesis	map00905	0	0	1	0	1	2	2	2
Terpenoid backbone biosynthesis	map00900	0	0	1	0	3	0	2	4
Flavonoid biosynthesis	map00941	0	0	2	0	1	0	0	4
Isoquinoline alkaloid biosynthesis	map00950	0	0	0	1	4	1	4	1
Glutathione metabolism	map00480	0	0	5	0	8	3	4	7

Up, the number of up-regulated genes; Down, the number of down-regulated genes.

### DEGs involved in defense response to *P*. *capsici*

**Fig 6:**
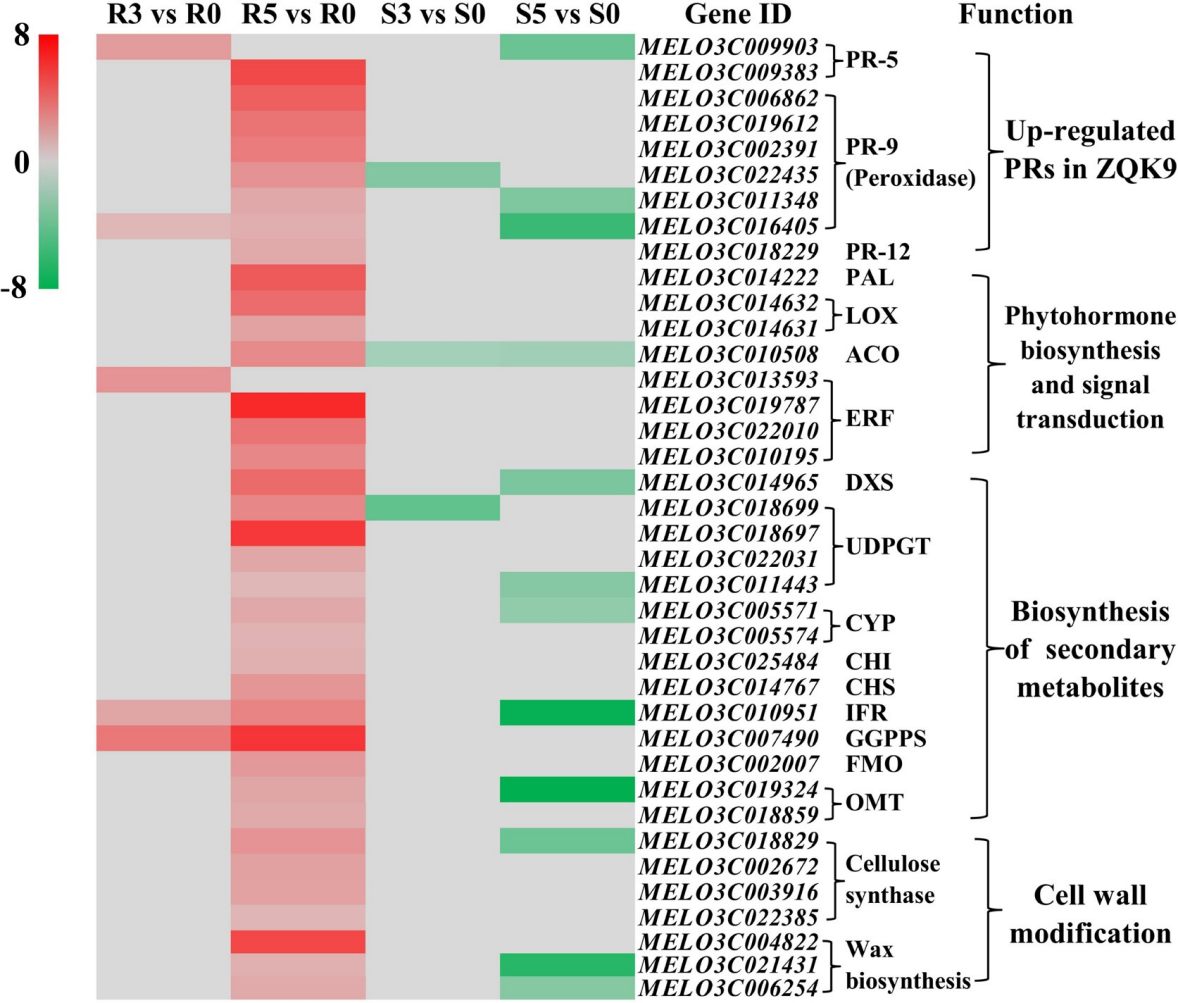
Hierarchical clustering of DEGs likely involved in defense response to *P*. *capsici* in ZQK9. Up-regulation and down-regulation were represented by red and green shading, respectively. Gray shading indicated non-modulation. R3 vs R0 and R5 vs R0 were the DEGs at 3 and 5 dpi in ZQK9, respectively. S3 vs S0 and S5 vs S0 were the DEGs at 3 and 5 dpi in E31, respectively. PRs, Pathogenesis-related proteins; PAL, Phenylalanine ammonia lyase; LOX, Lipoxygenase; ACO, 1-aminocyclopropane-1-carboxylate oxidase; ERF, Ethylene-responsive transcription factor; DXS, 1-Deoxy-d-xylulose 5-phosphate synthase; UDPGT, UDP-glycosyltransferase; CYP, Cytochrome P450; CHI, chalcone flavonone isomerase; CHS, chalcone synthase; IFR, isoflavone reductase; GGPPS, geranylgeranyl pyrophosphate synthase; FMO, flavin-containing monooxygenase; OMT, O-methyltransferases.

Based on the functional annotations, 38 DEGs may be involved in defense response to *P*. *capsici* in ZQK9, including 9 PR genes, 14 secondary metabolites biosynthesis genes, 7 cell wall modification genes and 8 hormones biosynthesis and signal transduction related genes (Fig 6).

### Validation of RNA-seq data by qRT-PCR

To confirm the reliability of RNA-seq results, 14 DEGs were selected for qRT-PCR analysis (S1 Table). Based on the functional annotations provided in previous reports, homologous genes of these DEGs were all found to be associated with the defense response to pathogen infection in different hosts. Generally, the expression profiles detected by qRT-PCR were consistent with the RNA-seq findings at all infection stages, suggesting that the RNA-seq results were highly reliable (S1 Fig).

## Discussion

*Phytophthora blight* caused widespread and devastating damage to melon industry. To date, studies of melon resistance to this disease mainly focused on the screening of highly resistant germplasm resources [7,8]. The resistant genes and molecular mechanisms were still unclear. The melon line ZQK9 was identified to be highly resistant to *P*. *capsici*, with no visible symptoms after artificial inoculation. PAS stain of inoculated roots showed that cell structure of ZQK9 remained intact, whereas in susceptible E31, the epidermal and cortical parenchyma cells of infected roots were ruptured at 5 dpi. It implied that the development of *P*. *capsici* hyphae was obviously inhibited in ZQK9. Thus, ZQK9 showed higher level of resistance to avoid the development of symptoms. But the differences between ZQK9 and E31 on inducible defense response against *P*. *capsici* infection were still unclear.

In this study, RNA-seq technique was used to investigate the resistant response in ZQK9 and E31 against *P*. *capsici* at 0, 3, and 5 dpi. A total of 7,019 DEGs were detected between ZQK9 and E31. Among them, more DEGs were detected at 5 dpi than at 3 dpi in both lines. Similarly, in tomato inoculated with *Xanthomonas perforans*, there were more DEGs found at later stage (6 dpi) than at early stage (6 hpi) [21]. Based on the GO and KEGG analyses, DEGs involved in the pathways of plant hormone signal transduction, secondary metabolite biosynthesis and cell wall modification were significantly enriched. These DEGs may participate in the defense response to *P*. *capsici* infection.

### Up-regulation of PR proteins in ZQK9

PR proteins are synthesized in response to microbial attack and serve to limit the growth of pathogens. PRs accumulate in infected tissue and are induced systemically [22]. According to their molecular mass, isoelectric point, localization, and biological activity, PRs can be classified into 17 families [23]. In this study, 9 PR-like genes were specifically up-regulated in ZQK9 after inoculation with *P*. *capsici*, including 2 thaumatin-like protein (TLP) genes, 6 PR-9 genes and one PR-12 gene.

TLPs are a group of PR-5 proteins that are induced in plants for resistance to pathogens infection. Previous reports have demonstrated that the overexpressed TLP genes, *P23* and *CsTLP*, led to increased tolerance to the oomycetes *P*. *citrophthora* and *P*. *infestans* in transgenic orange and potato, respectively [24,25], suggesting the promising resistant function of TLP genes to oomycetes. In this study, one TLP gene *MELO3C009903* was significantly up-regulated in ZQK9 at 3 dpi, but contrarily down-regulated in E31 at 5 dpi. Another TLP gene *MELO3C009383* was specifically up-regulated in ZQK9 at 5 dpi, but not significantly induced in E31. The up-regulation of these two TLP genes may lead to the high resistance level in ZQK9 against *P*. *capsici* and further studies were needed to confirm.

PR-9 proteins have peroxidase activity and are involved in biosynthesis of ligin and suberin, which act as the cell wall barrier against pathogens. In ZQK9, 6 up-regulated peroxidase genes were enriched to GO:0009664 (plant-type cell wall organization) (Fig 6). Among these genes, *MELO3C016405* was annotated as peroxidase 72-like gene (*Prx72*) and up-regulated at 3 and 5 dpi in ZQK9, but down-regulated in E31 at 5 dpi. *MELO3C016405* was also enriched to GO:0009809, which indicated that this gene was related to lignin biosynthesis. In *Arabidopsis*, the down-regulation of *AtPrx72* led to disruption of the whole lignin biosynthesis route and caused thinner secondary walls in interfascicular fibers [26]. Hence, we predicted that the up-regulated *MELO3C016405* may be able to promote the cell wall strengthening in ZQK9.

PR-12 denoted as plant defensins are endogenous antimicrobial polypeptides that form an important component of the plant immune system. In transgenic tomato plants, over-expressing the pepper defensin gene *CaDef* enhanced the ability to inhibit *Fusarium sp*. and *P*. *infestans* [27]. Similarly, constitutive expression of *NmDef02* gene derived from *Nicotiana megalosiphon* in potato plants also delivered enhanced resistance against *P*. *infestans* [28]. In this study, *MELO3C018229* was specifically up-regulated in ZQK9 at 5 dpi. Homology analysis indicated that the MELO3C018229 protein possessed one glycine and eight cysteine residues, which were strictly conserved in many plant defensins including *CaDef* and *NmDef02*, implying that *MELO3C018229* may have antimicrobial function (S2 Fig).

### Phytohormone biosynthesis and signal transduction

Salicylic acid (SA), jasmonic acid (JA) and enthylene (ET) are typical phytohormones involved in the activation of plant defense against pathogens. Previous reports suggested that SA signaling was associated with the activation of resistance to biotrophic phytopathogen, while ET and JA were involved in the resistance to necrotrophs [29]. *Phytophthoras* spp. are hemi-biotrophic pathogens in nature, therefore, the mechanism of host resistance induced by hormones is more complex [30]. Shah SRA et al. reported that ET and SA, but not JA, were involved in the late blight resistance in tomato [31]. Wu et al. found that both SA and JA/ET dependent signaling pathways were important in *Nicotiana benthamiana* defenses against *P*. *nicotianae* [32].

Phenylalanine ammonia lyase (PAL) was reported as an important enzyme in SA biosynthesis [33]. In this study, PAL gene *MELO3C014222* was significantly up-regulated in ZQK9 at 5 dpi. Thus, it may have a positive effect on SA-induced defense responses.

Lipoxygenase (LOX) was the key enzyme catalyzing JA biosynthesis [34]. In this study, *MELO3C014631* and *MELO3C014632* were enriched to the GO:0016165 term (linoleate 13-lipoxygenase activity, 13-LOX) and specifically up-regulated in ZQK9 at 5 dpi. In pepper, *P*. *capsici* infection also induced the up-regulation of 13-LOX homologous gene *CaLOX2* [35]. In transgenic tomato, overexpression of the 13-LOX gene *TomloxD* increased the generation of JA and enhanced the plant resistance level to *Cladosporium fulvum* [36].

1-aminocyclopropane-1-carboxylate oxidase (ACO) plays an important role in ethylene synthesis [37]. After *P*. *capsici* inoculation, the ACO gene *MELO3C010508* was up-regulated in ZQK9 at 5 dpi, but down-regulated in E31 at 3 and 5 dpi. ERFs governed the ethylene signaling and played positive regulatory roles in initiating downstream defense response genes, such as *Pti4-6* in tomato [38], *AtEBP* in *Arabidopsis* [39], and *OsRap2*.*6* in rice [40]. In this study, 4 ERF genes, *MELO3C010195*, *MELO3C013593*, *MELO3C019787*, and *MELO3C022010*, were specifically up-regulated in ZQK9. Thus, the induction of these genes indicated that ethylene may be involved in the resistance to *P*. *capsici* in ZQK9.

### Biosynthesis of secondary metabolites

Isoprene is the basic element of terpenes which constitute the largest class of secondary metabolites in plants [41]. 1-Deoxy-d-xylulose 5-phosphate synthase (DXS) catalyzes the initial step of isoprenoid biosynthesis. The up-regulation of *DXS* genes have proved to be relevant in enhancing defense against biotic stresses. In poplars, overexpression of *PtDXS* enhanced the resistance to *Septotis*. *populiperda* infection [42]. Similarly, in tomato, overexpression of *SlMX1* increased the plant tolerance to *P*. *infestans* and *Botrytis cinerea*, which caused late blight and gray mold, respectively [43]. In this study, one DXS synthase gene *MELO3C014965* was significantly up-regulated in ZQK9 but down-regulated in E31 at 5 dpi. So we inferred that this gene may play a positive role in the defense response to *P*. *capsici* in ZQK9.

Flavonoids are another group of secondary metabolites widely present in plants, and they also play essential roles in plant defense against biotic stress [44]. In this study, 13 DEGs were annotated as the key catalytic enzymes in flavonoids biosynthesis and they were significantly up-regulated in ZQK9, but down-regulated or not significantly induced in E31 (Fig 6). Among them, 5 DEGs were homologous to 6 genes of cucumber cultivar ‘Vlaspik’, which performed an age-related resistance (ARR) to *P*. *capsici*. To be specific, *MELO3C002007* was homologous to *Csa3G033770 and Csa3G033780*, which functioned as flavine-containing monooxygeneaes (FMO). *MELO3C010951* was homologous to isoflavone reductase (IFR) genes *Csa7G002520* and *Csa7G004020*. *MELO3C007490* was homologous to *Csa7G211090* with the function of geranylgeranyl pyrophosphate synthase (GGPPS). *MELO3C018859* and *MELO3C019324* were homologous to *Csa7G039280*, which acted as O-methyltransferases (OMT). These cucumber genes were significantly up-regulated in the fruit peel of resistant ‘Vlaspik’ at 16 days post pollination after *P*. *capsici* inoculation and were considered to be associated with age-related resistance [10].

### Cell wall modification

Reinforcing the cell wall to prevent further pathogen ingress is the most common response of the infected host plant. Cell wall enhancement is always achieved by cellulose synthesis, lignification, and callose deposition [45]. Histochemical staining showed that there were no significant changes in the cell morphology of ZQK9 after *P*. *capsici* infection, while the epidermal cells of E31 were disintegrated (Fig 2). Based on GO annotation, the GO terms related to cell wall modification were significantly enriched in ZQK9. The up-regulated PR-9 genes mentioned above may be related to cell wall lignification. Another 4 genes (*MELO3C002672*, *MELO3C003916*, *MELO3C018829* and *MELO3C022385*) involved in cellulose synthesis were also specifically up-regulated in ZQK9 after *P*. *capsici* inoculation. Thus, these induced genes may play key roles in strengthening the cell wall of ZQK9 against *P*. *capsici* ingression. Cuticular wax, adcrusted on the epidermis of plant organs, served as the physical barrier to protect the plant from pathogens attack. In this study, 3 DEGs were enriched to the wax biosynthesis pathway and up-regulated in ZQK9. *MELO3C004822* and *MELO3C021431* were annotated as fatty acyl-CoA reductase 3 (FAR3), which was involved in long chain fatty alcohol synthesis for cuticular wax formation [46]. *MELO3C006254* was homologous to *ECERIFERUM 1* (*CER1*). Mark et al. reported that the *CER1* gene of *Arabidopsis* was involved in epicuticular wax biosynthesis [47].

## Conclusions

In this study, RNA-seq was used to analyze the root transcriptome of resistant ZQK9 and susceptible E31 after *P*. *capsici* infection. The results provided a perspective on the expression patterns of defense-related genes involved in melon-*P*. *capsici* interactions. Several candidate genes were identified and will contribute to future studies in uncovering the underlying molecular mechanisms of melon resistance to *P*. *capsici*.

## Supporting information

S1 Fig qRT-PCR validation of 14 DEGs related to defense response at 3 and 5 dpi. Gene expression was normalized to actin. Data was displayed as the mean ± SD of three biological replicates. The x-axes represented the names of sequencing libraries and the y-axes indicated relative fold change value. The asterisk above the bars indicated statistically significant differences between the infected samples and corresponding control samples. Significance levels were indicated as * p < 0.05 and ** p < 0.01.
(TIF)

S2 Fig Homology analysis of MELO3C018229, CaDef and NmDef02 protein sequences. Red asterisks indicated one glycine and eight cysteine residues conserved in defensin protein sequences.
(TIF)

S1 Table Genes and primers used in qRT-PCR experiments. (DOC)

S2 Table Summary of RNA-seq data. (DOC)

S3 Table Summary of mapped reads. (XLS)

S4 Table Mapping region statistic. (XLS)

S5 Table Hierarchical clustering of differentially expressed genes among four groups, R3 VS R0, R5 VS R0, S3 VS S0 and S5 VS S0. (XLS)

S6 Table Gene ontology (GO) analysis of differentially expressed genes for biological process in ZQK9 and E31 at 5 dpi. (XLS)

S7 Table KEGG classifications of DEGs specific to ZQK9 and E31 inoculated with *P*. *capsici*. (XLS)
