# Supplementary figures and images for: Transcriptome analysis clarified genes involved in resistance to Phytophthora capsici in melon
Source: PLoS One. 2020 Feb 12;15(2):e0227284. doi: 10.1371/journal.pone.0227284 (PMC7015699; doi:10.1371/journal.pone.0227284)

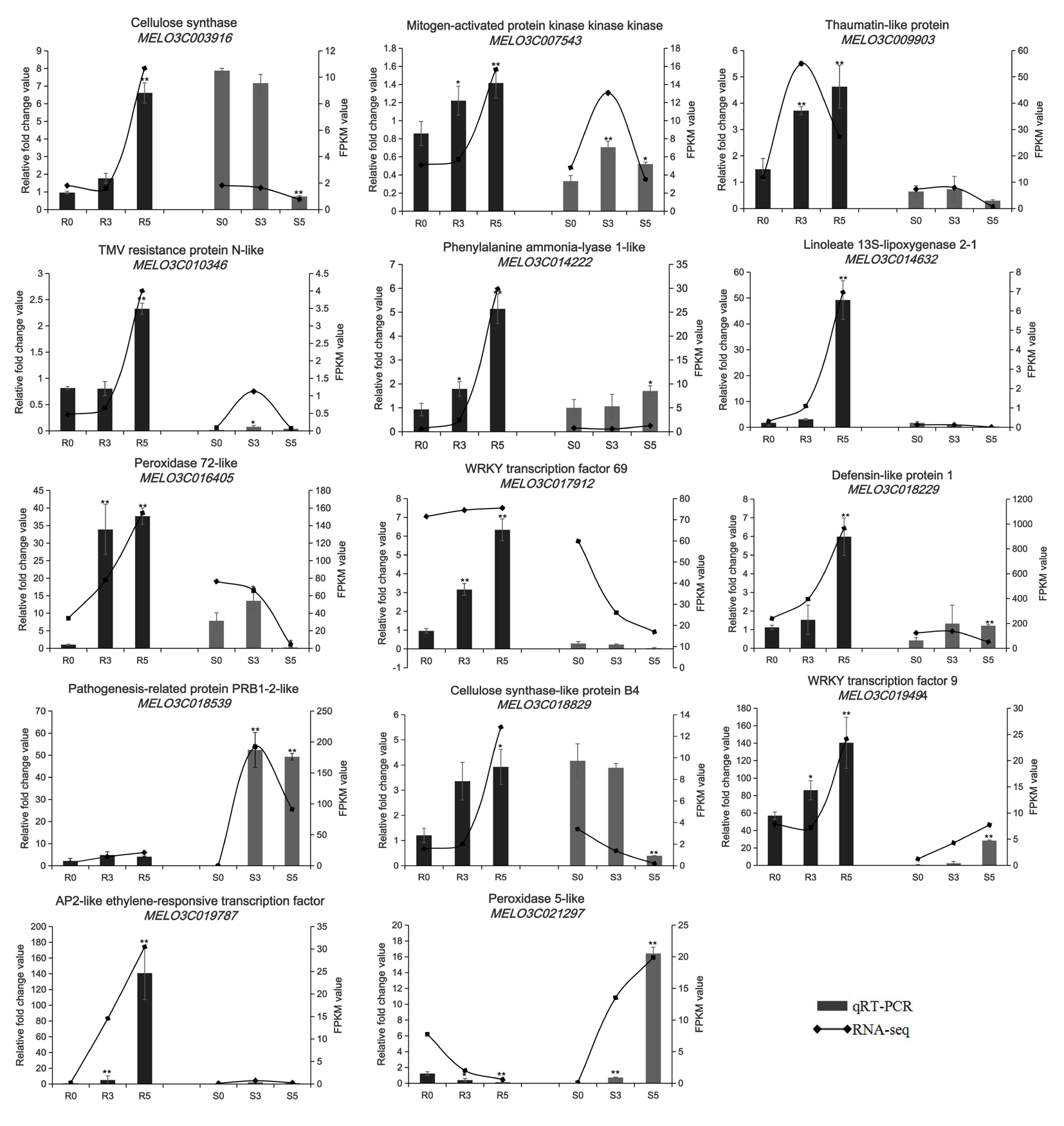

Supplement: S1 Fig — Gene expression was normalized to actin. Data was displayed as the mean ± SD of three biological replicates. The x-axes represented the names of sequencing libraries and the y-axes indicated relative fold change value. The asterisk above the bars indicated statistically significant differences between the infected samples and corresponding control samples. Significance levels were indicated as * p < 0.05 and ** p < 0.01. (TIF) [file pone.0227284.s001.tif]

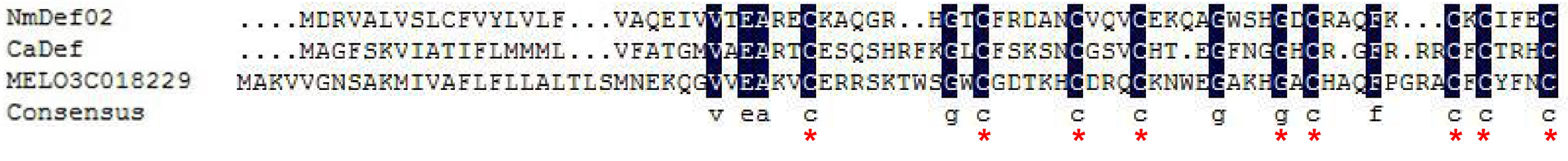

Supplement: S2 Fig — Red asterisks indicated one glycine and eight cysteine residues conserved in defensin protein sequences. (TIF) [file pone.0227284.s002.tif]
