# Supplementary material for: Transcriptome analysis clarified genes involved in resistance to Phytophthora capsici in melon
Source: PLoS One. 2020 Feb 12;15(2):e0227284. doi: 10.1371/journal.pone.0227284 (PMC7015699; doi:10.1371/journal.pone.0227284)
Supplement: S1 Table — (DOC) [file pone.0227284.s003.doc]

**S1 Table.** **Genes and primers used in qRT-PCR experiments.**

| **Gene** | **Gene Function** | **Primers (5’ to 3’)** |
| --- | --- | --- |
| *MELO3C003916* | Cellulose synthase | F: TGTCGCTGGGTCTCATAATCG |
|  |  | R: CATCCACAGTAAGCCCTACTTCA |
| *MELO3C007543* | Mitogen-activated protein kinase kinase kinase | F: GTGGAATTGTTTGGCGTTTTG |
|  |  | R: GGAGACAGGGTCAGGCGTAGT |
| *MELO3C009903* | Thaumatin-like protein | F: ATCACCCTCCAAGAAGACAAATC |
|  |  | R: GAACGAGTCCAATCCAAAAGC |
| *MELO3C010346* | Protein suppresspr of npr1-1 | F: AAGATACATCAGCAAGTCAGGGTC |
|  |  | R: GGGATTCATACTTGAGGTGGC |
| *MELO3C014222* | Phenylalanine ammonia-lyase | F: ATTTTGTCGGGCATCTTTG |
|  |  | R: GCGATCTTGTTTTGGCTTCT |
| *MELO3C014632* | Linoleate 13S-lipoxygenase 2-1 | F: AACGCCTTTCGCTGCTT |
|  |  | R: TGTAGGACTCTGGTGGTGGA |
| *MELO3C016405* | Peroxidase 72-like | F: AACAACACTTTCCAAACCATTCTC |
|  |  | R: CATCGCTTCCGCAACTCG |
| *MELO3C017912* | WRKY transcription factor 69 | F: AAAATGAAGGTCCACCTCCTG |
|  |  | R:GATGGTTATGGCTTGAAGTGTATG |
| *MELO3C018229* | Defensin-like protein 19 | F: CCTTTTGGCCCTCACGC |
|  |  | R: TCCATGTTTCGCACCTTCC |
| *MELO3C018539* | Pathogenesis-related protein PRB1-2-like | F: CGAACCAACGCATCAACG |
|  |  | R: CACCTTACCAGCCGCACA |
| *MELO3C018829* | Cellulose synthase-like protein B4 | F:TACCCTCAACACTTCAAACAAGTACC |
|  |  | R: CCCCATCGTCGGAGACATAG |
| *MELO3C019494* | WRKY transcription factor 9 | F: CCTCCTCAACCTCCCACTTC |
|  |  | R: TGGCAGACACGGCATCG |
| *MELO3C019787* | AP2-like ethylene-responsive transcription factor | F: CTTCGTTTTCCTATCTTCCAATCC |
|  |  | R: CATCAACAAAGTCAAGTAGCCCTC |
| *MELO3C021297* | Peroxidase 5-like | F: GACTGCTTTGTTAGGGGATGC |
|  |  | R: CAATTTTCGCCTTGGCTTC |
| [*MELO3C023264*](http://cucurbitgenomics.org/feature/gene/MELO3C023264) | Actin | F: CCTGGTATCGCTGACCGTAT |
|  |  | R: TACTGAGCGATGCAAGGATG |
