# Supplementary material for: Transcriptome analysis clarified genes involved in resistance to Phytophthora capsici in melon
Source: PLoS One. 2020 Feb 12;15(2):e0227284. doi: 10.1371/journal.pone.0227284 (PMC7015699; doi:10.1371/journal.pone.0227284)
Supplement: S2 Table — (DOC) [file pone.0227284.s004.doc]

**S2 Table.** **Summary of RNA-seq data.**

| **Library** | **No. of raw reads** | **Clean reads** | | **Mapped reads** | | **No. of matched genes** |
| --- | --- | --- | --- | --- | --- | --- |
|  |  | **Total** | **Raw reads**  **(%)** | **Total** | **Clean reads**  **(%)** |  |
| R0-1 | 46,643,822 | 45,112,342 | 96.72% | 37,686,512 | 83.54% | 19,834 |
| R0-2 | 47,967,708 | 46,318,048 | 96.56% | 38,439,610 | 82.99% | 19,744 |
| R0-3 | 48,648,770 | 46,982,868 | 96.58% | 39,604,029 | 84.29% | 19,842 |
| R3-1 | 48,459,704 | 46,801,206 | 96.58% | 38,616,884 | 82.51% | 19,832 |
| R3-2 | 46,513,410 | 44,821,348 | 96.36% | 37,360,366 | 83.35% | 19,904 |
| R3-3 | 40,986,962 | 39,801,904 | 97.11% | 34,346,694 | 86.29% | 19,835 |
| R5-1 | 41,642,012 | 40,235,376 | 96.62% | 33,975,957 | 84.44% | 19,611 |
| R5-2 | 44,678,180 | 43,255,742 | 96.82% | 36,678,499 | 84.79% | 20,155 |
| R5-3 | 41,286,172 | 39,898,358 | 96.64% | 33,719,608 | 84.51% | 20,203 |
| S0-1 | 45,909,018 | 44,289,860 | 96.47% | 37,183,649 | 83.96% | 19,341 |
| S0-2 | 47,379,332 | 45,921,352 | 96.92% | 39,211,440 | 85.39% | 19,741 |
| S0-3 | 45,735,194 | 43,875,724 | 95.93% | 36,119,168 | 82.32% | 19,525 |
| S3-1 | 40,811,306 | 39,661,576 | 97.18% | 31,360,486 | 79.07% | 18,650 |
| S3-2 | 42,181,840 | 40,726,156 | 96.55% | 34,779,349 | 85.40% | 19,548 |
| S3-3 | 48,816,652 | 47,172,082 | 96.63% | 40,040,109 | 84.88% | 19,473 |
| S5-1 | 47,158,316 | 45,668,328 | 96.84% | 37,132,824 | 81.31% | 19,479 |
| S5-2 | 41,229,168 | 40,028,936 | 97.09% | 32,680,468 | 81.64% | 18,404 |
| S5-3 | 40,061,674 | 38,620,084 | 96.40% | 31,527,823 | 81.64% | 18,613 |
